# Study of the Corrosion Resistance of Austenitic Stainless Steels during Conversion of Waste to Biofuel

**DOI:** 10.3390/ma10030325

**Published:** 2017-03-22

**Authors:** Marina Cabrini, Sergio Lorenzi, Tommaso Pastore, Simone Pellegrini, Mauro Burattini, Roberta Miglio

**Affiliations:** 1 Department of Engineering and Applied Sciences, University of Bergamo, Dalmine (BG) 24044, Italy; marina.cabrini@unibg.it (M.C.); Tommaso.pastore@unibg.it (T.P.); simone.pellegrini@unibg.it (S.P.); 2 Consorzio INSTM, Via G. Giusti 9, Firenze 50121, Italy; 3 Eni SpA, Renewable Energy & Environmental Laboratories Istituto Donegani, Via G. Fauser 4, Novara (NO) 28100, Italy; mauro.burattini@eni.com (M.B.); roberta.miglio@eni.com (R.M.)

**Keywords:** corrosion, stress corrosion cracking, bio-fuel, stainless steels, liquefaction, sustainability, organic fraction of solid municipal waste, bio-oil

## Abstract

The paper deals with the corrosion behavior of stainless steels as candidate materials for biofuel production plants by liquefaction process of the sorted organic fraction of municipal solid waste. Corrosion tests were carried out on AISI 316L and AISI 304L stainless steels at 250 °C in a batch reactor during conversion of raw material to bio-oil (biofuel precursor), by exposing specimens either to water/oil phase or humid gas phase. General corrosion rate was measured by weight loss tests. The susceptibility to stress corrosion cracking was evaluated by means of U-bend specimens and slow stress rate tests at 10^−6^ or 10^−5^ s^−1^ strain rate. After tests, scanning electron microscope analysis was carried out to detect cracks and localized attacks. The results are discussed in relation with exposure conditions. They show very low corrosion rates strictly dependent upon time and temperature. No stress corrosion cracking was observed on U-bend specimens, under constant loading. Small cracks confined in the necking cone of specimens prove that stress corrosion cracking only occurred during slow strain rate tests at stresses exceeding the yield strength.

## 1. Introduction

The development of the green economy is based on an industrial model in discontinuity with the past and the idea that the entire production chain must be sustainable and integrated into the local urban fabric. According to this approach, the use of organic waste as a renewable energy source is surely a topical issue. In Italy, municipal solid waste (MSW) and green crops in 2012 stood at 4.8 million tons. In the regions where recycling is effectively applied, the collection of bio-waste reaches 45–60 kg/inhabitant/year.

Biofuels coming from waste feature a double benefit, being the result of a process of waste recovery, which is preferable to disposal according to the management hierarchy prescribed by the regulations, and it is not in competition with the food industry, as in the case of the first generation biofuels.

The thermochemical conversion of organic waste into biofuel by a liquefaction process has been developed by Renewable Energy and Environmental R&D Center (ENI-Donegani) in Novara. The results of this research project led to the realization of batch laboratory equipment and a pilot scale prototype for continuous operation [1,2,3,4].

The corrosion risk assessment of the materials and equipment operating in contact with such an environment represents a crucial step. Bio-oils and processing fluids can contain significant concentrations of aggressive substances derived from organic waste conversion, but there is currently a lack of data. Few works have been published on the evaluation of the corrosion resistance of traditional steels in contact with bio-oils produced from biomass; moreover, none of them refers to the specific operating conditions of the liquefaction processes considered in this paper.

According to Keiser et al. [5], carbon steels or low alloyed steels containing chromium and molybdenum show corrosion rates ranging from less than 1 mm/year up to 4 mm/year in bio-oil at 50 °C, depending upon Total Acid Number (TAN). They report corrosion rates between 0.1 and 0.8 mm/year on an AISI 409 ferritic stainless steel—containing about 11.5% of chromium—and negligible corrosion rates on austenitic stainless steels with 18% chromium without molybdenum (AISI 304) and with molybdenum (AISI 316).

Aubin and Roy [6] outlined the increase of bio-oils corrosivity with temperature, strictly dependent upon oil on water ratio. The presence of formic and acetic acids causes a decrease in pH of bio-oils produced by pyrolysis to values in the range of 2–3 [7]. The aggressiveness increases particularly for carbon steels and aluminum alloys rather than for stainless steels.

Hu et al. [8] carried out tests in bio-oil obtained by pyrolysis containing 30% of water, at temperatures between 25 and 50 °C. The pH was in the range 3–3.5 due to the presence of organic acids. They observed high corrosion rates on lead and iron, slightly lower on AISI 1045 carbon steel, whist copper did not show general corrosion [8,9]. In a previous work, Darmstadt et al. [9] report on bio-oils from vacuum pyrolysis of bark residues at 80 °C. They outline that the significant amount of acidic water in the bio-oils caused severe corrosion of aluminum, to a less extent of copper, while the corrosion of AISI 316 stainless steel was substantially nil.

In the temperature range of interest for liquefaction process—between 240 and 310 °C—there is a lack of data on the behavior of austenitic stainless steels in bio-oils with high TAN, since pyrolysis bio-oils tend to decompose and polymerize. Most of the available information regarding the corrosivity induced by organic acids at high temperatures comes from the experience of the refineries. According to Nagi-Hanspal et al. [10], oils with TAN higher than 0.5 mg KOH/g bio-oil or distilled fractions with TAN greater than 1.5 are potentially corrosive at temperatures between 230 and 400 °C. In the refinery practice, despite the value of the TAN including all acids in crude oil, TAN is typically associated with the content of naphthenic acids, since they are primarily responsible for high temperature corrosion. Corrosion by naphthenic acid is controlled by using stainless steels containing molybdenum, mainly AISI 316L, AISI316Nb, and AISI 317.

Keiser et al. [11] report that pyrolysis oil causes cracking in carbon and low alloy steels. They investigated the risk of stress corrosion cracking (SCC) by exposing U-bend specimens in pyrolysis oil at 50 °C. Cracks were found on carbon and low alloy steels after 250-h exposure. Shallow cracks were also found on stainless steels after 500 and 750 h exposures. Furthermore, micro-cracks were observed on austenitic stainless steel and nickel alloys exposed directly in the pyrolysis plant at the output of the fluids, at a temperature of 550 °C. The presence of chloride induced SCC was also demonstrated by failure analysis of components exposed to contact with corrosive bio-oils [5,12,13].

The liquefaction process involves the thermochemical conversion of sorted organic fraction of MSW in bio-oil. Before feeding in the reactor, the raw material is crushed until a particle size less than 2 mm is attained and then it is homogenized. In the reactor, the charge undergoes a heat treatment at temperatures between 240 and 310 °C, for durations ranging from 0.5 up to 4 h and pressures from 40 up to 110 bar, due to liquid vapor pressure and the formation of non-condensable gases.

The conversion takes place with a multi-phase process for the simultaneous formation of a gaseous phase, rich in acid gases (mainly CO_2_, H_2_S), an aqueous phase containing inorganic compounds—such as chlorides and sulfates—and organic compounds, an oil phase—that is the bio-oil—and a solid phase constituted by carbonaceous and inorganic residues.

The oil phase is a mixture of fatty acids and their amides, aromatics, paraffins and olefins, ketones, alkyl cycloalkanes or cycloalkenes, alkylated pyrrolidones, methyl or ethyl esters of fatty acids, alkylated furans, steroids, etc. More detailed description was published in previous papers by Leonardis et al. and Chiaberge et al. [14,15]. During the reaction, homogeneous system that separates during the cooling forms. The oil (after reparation of the solid phase with the aid of a solvent—ethylacetate) contains a total amount of water lower than 0.1% with dissolved inorganic compounds, which are mainly constituted of sodium salts. The TAN is influenced by the characteristics of the charge and by the operating conditions of liquefaction. Chiaberge et al. [16] measured on bio-oils derived from waste food typical TAN values between 20 and 150. After cooling, the pH of aqueous phase is between 6 and 8.5. Its composition is complex. The analysis indicates the presence of organic acids (i.e., formic, acetic, propionic, butyric, levulinic acids), sugars, and alcohols as well as inorganic salts. Inorganic anions includes chlorides (about 4000–5000 ppm), sulphur ions, and phosphorus ions. Among cations, sodium, potassium, iron, calcium, and magnesium were mainly detected. Inorganic carbon is also found in many different chemical combinations, including hydrated carbon dioxide, bicarbonate, and carbonate, depending on pH.

The solid residue consists of inorganic elements, in which Ca and P prevail, probably due the precipitation of insoluble salts (i.e., phosphates and mixed carbonates of Ca and Mg) derived from bones present in the feed. Furthermore, organic fraction is still present in the solid residue, as demonstrated by not negligible calorific value. More details on the chemical-physical characterization of the phases produced by liquefaction on organic fraction of MSW are reported elsewhere [17].

The aim of the work is the study of the corrosion and stress corrosion cracking behavior of traditional AISI 316L and 304L austenitic stainless steels exposed to the environmental conditions occurring during liquefaction process of organic sorted fraction of municipal solid waste.

## 2. Results

**Figure 1:**
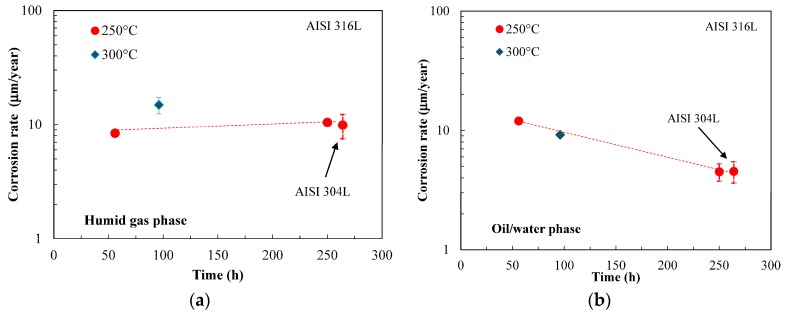
Weight loss measurements to evaluate the effect of time on the corrosion rate of AISI 316L steel during SMSW liquefaction at 250 °C. (**a**) In humid phase; (**b**) in oil/water phase.

**Figure 2:**
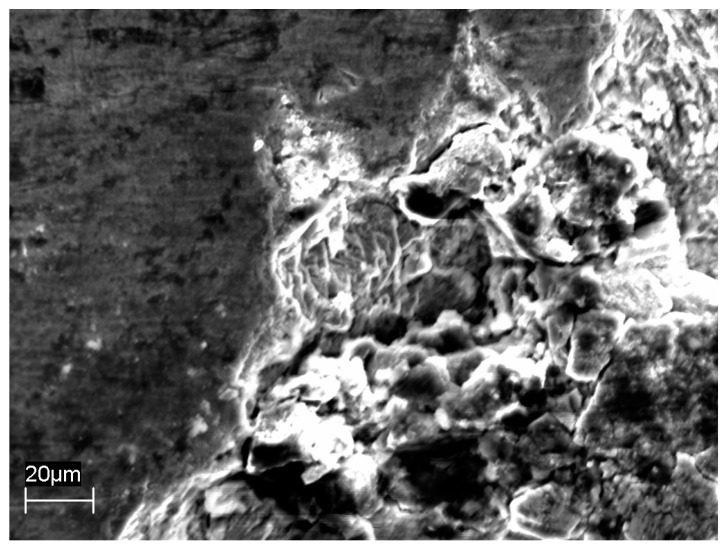
Shallow pit on AISI 304L steel after SMSW liquefaction at 250 °C for 264 h.

Figure 1 shows the corrosion rates estimated by weight loss measurements on U-bend specimens after tests performed during the SMSW liquefaction process at different temperatures, for durations ranging from 55 to 264 h. The corrosion rate is calculated as mean values on exposed surface. The general attack is low, with rates in the humid gas phase that remains constant over the testing time at about 10 μm/year. At 300 °C, the values slightly rise. In the oil/water phase, the corrosion rate shows values comparable to that in gas phase, but the rate tends to decrease with time approaching at about 5 μm/year. The tests on AISI 304L steel were only executed at 250 °C. AISI 316L and AISI 304L steels showed similar corrosion rates (Figure 1), but shallow and widened sporadic pits were observed on the surface of AISI 304L stainless steel (Figure 2).

**Figure 3:**
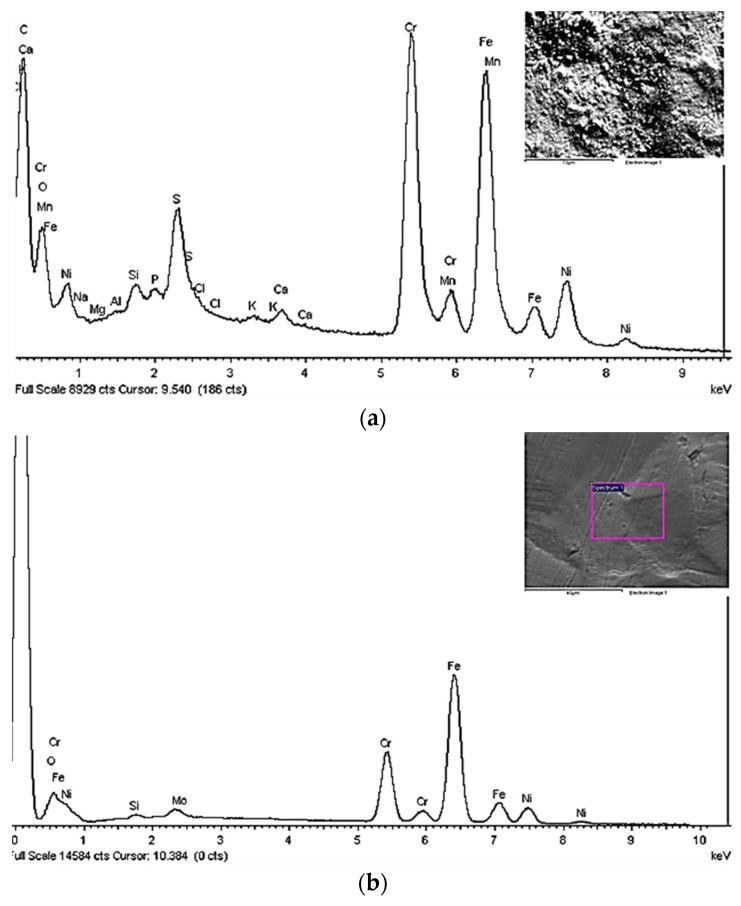
EDS analysis of the oxide film formed on AISI 316L U-Bend specimens (**a**) and base material composition as reference (**b**).

After testing, all U-bend specimens showed a dyed layer of oxide below a carbonaceous scale, with colours varying from metallic grey to black-brown, passing through bronze, gold, red, purple, blue, and green. The change of colour denotes an increase in film thickness, considering the interference nature of film colours on stainless steel. The Energy Dispersive Spectroscopy (EDS) analysis showed superficial depletion of iron and an increase in chromium and oxygen, indicating that the oxide layer was mainly constituted of chromium oxide (Figure 3).

**Figure 4:**
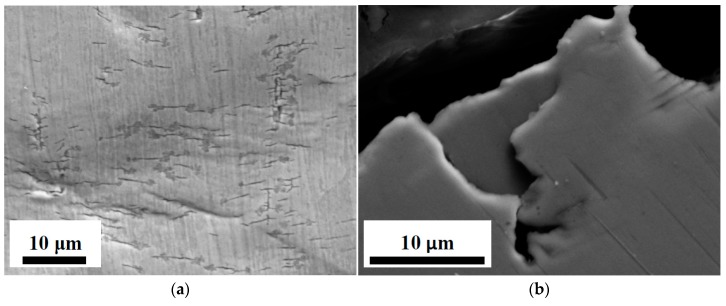
SEM image of micro-defect on AISI 316L U-bend specimens after test at 250 °C for 55 h: external surface (**a**); metallographic section (**b**).

Visual examinations of exposed surfaces were performed and metallografic sections of U-bend specimens were carried out in order to assess stress corrosion cracking. The specimens did not show any cracking. Only micro-defects were observed on slip bands with depth limited to 20 μm (Figure 4).

**Figure 5:**
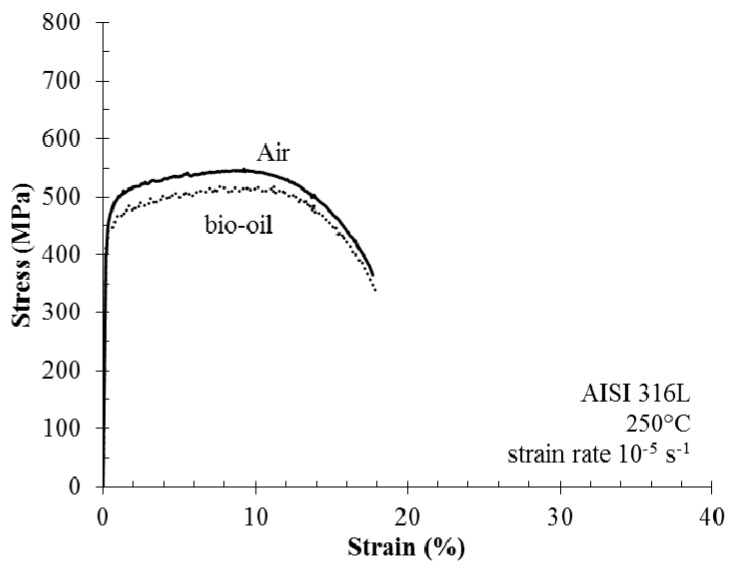
Stress vs. strain curves of AISI 316L in air and in autoclave during bio-oil conversion.

**Figure 6:**
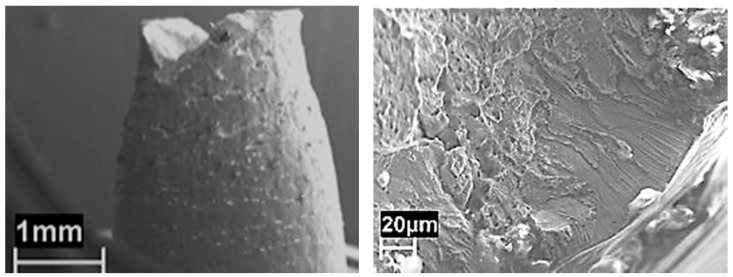
SEM images of fracture morphology of AISI 316L steel after SSR tests in autoclave, during bio-oil conversion at 250 °C at 10^−6^ s^−1^.

Figure 5 compares the stress/strain curve collected during SSR tests in the batch reactor to the corresponding curve in air at 10^−5^ s^−1^ strain rate. The tests in the batch reactor were performed during the liquefaction reaction. Not relevant differences can be underlined. Nevertheless, the specimens exposed during liquefaction reaction show numerous shallow cracks. The fracture originates from the coalescence of these cracks on the surface of the specimen, producing an appreciable decrease of ductile parameters, mainly the reduction of area. The cracks have a brittle growth featuring the typical morphology of chloride SCC (Figure 6). Their growth on the fracture surface is limited to less than 150 μm. Secondary cracks were also present on the surface of the necking cone, with similar morphology. Some micro-cracks have also been found far from the necking due to work hardening induced by the machining of specimens.

**Figure 7:**
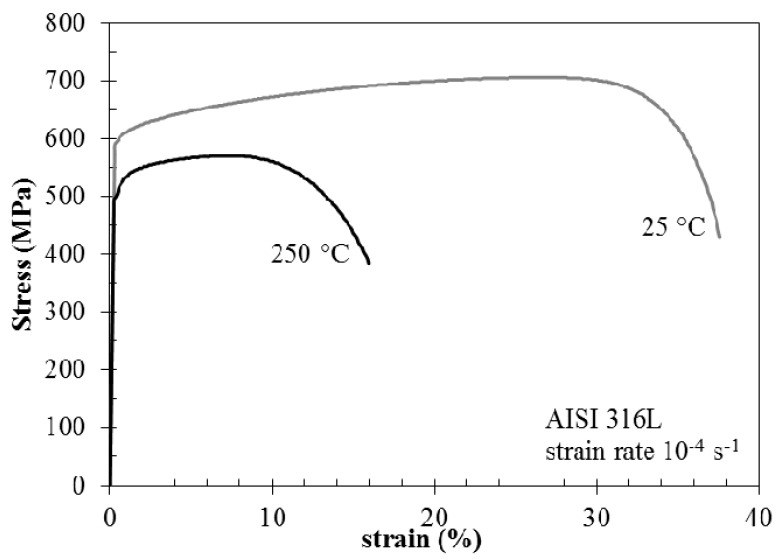
Effect of temperature on the stress vs. strain curves of AISI 316L in air.

At 250 °C, the stress/strain curves showed a noticeable reduction of strength—both tensile yield stress and ultimate tensile stress—compared with the behaviour at room temperature (Figure 7). Furthermore, the ductility also decreases with a significant reduction of elongation to fracture, but with a less extensive variation of the reduction of area. Byun et al. [18] reports on temperature dependence of strain hardening of austenitic stainless steels and plastic instability occurrence. Strain hardening of austenitic stainless steels at high temperature promotes the early insurgence of necking, thus limiting the extension of the uniform plastic deformation. Actually, the fracture surface of the specimens tested in air is fully ductile in any case. At 25 °C, the fracture surface has typical ductile morphology with small dimples. At 250 °C, the dimples appear larger and transversal ductile separations are visible.

In order to assess the extent of SCC phenomena during SSR tests, embrittlement indexes were calculated by using elongation at break (IA%) and reduction of area (IZ%). The ratio between the value of the parameter in environment and the corresponding value in air was determined according to the following relationships:

(1) $$\mathrm{IA}\%\;=\left(1-\frac{A_{\%\;environment}}{A_{\%\;air}}\right)\times 100$$

(2) $$\mathrm{IZ}\%\;=\left(1-\frac{Z_{\%\;environment}}{Z_{\%\;air}}\right)\times 100$$

The evaluation of the SCC indexes is based on the increase of the value above zero.

Table 1 summarizes the results of SSR tests. They indicate a general low susceptibility to SCC for the AISI 316L stainless steel, being in the range of 10%–27%. Minor differences can be noticed between different testing conditions. According to the fracture analysis of the specimens, the authors decided to use the IZ% index rather than IA% to discuss the effect of environmental conditions on SCC. The test performed on AISI 304 steel confirms slightly worse behaviour compared to AISI 316 steel.

## 3. Discussion

The results confirm that the two austenitic stainless steels are substantially resistant to corrosion (both localized corrosion and SCC) under the environmental conditions of batch liquefaction process The very low corrosion rates measured during tests should be considered negligible in any case. Actually, traditional austenitic stainless steels as AISI 316L and AISI 304L steels have high general corrosion resistance due to the formation of passive film made of chromium-rich oxide. The protective film is stable in a wide range of pH values of the liquid phase.

Despite the high chloride concentration of the water phase, in the range between 4000–5000 mg/L, and high temperature (240–300 °C), pitting corrosion was not clearly noticeable. The initiation of pitting was only sporadic, and limited to AISI 304L steels, without any significant propagation. Pitting generally occurs in the presence of oxidant, under conditions that promote corrosion potentials sufficiently noble to overcome the pitting potential. The corrosion potential of passive stainless steel mainly depends on the oxidizing power of the environment. It is considerably lower in reducing conditions than in oxidizing ones. Otherwise, the pitting potential depends on chlorides concentration, pH, and temperature of solution.

The batch process produces low corrosivity due to its short duration and modifications of environmental parameter during the early period of liquefaction reactions. In the early stages of the batch process of liquefaction, the presence of dissolved oxygen in the charge produces an oxidizing environment favourable to pit initiation. However, at sufficiently high temperatures reducing conditions develop that lower the corrosion potential, reducing the risk of localized corrosion. Thus, the most critical conditions are primarily present in the initial transient stage. Then the oxygen depletion also reduces the propagation of shallow pits initiated during early period. It is quite difficult to measure the dissolved concentration of O_2_ in batch liquefaction, due to the high level of temperature and pressure.

During thermal treatment, different reactions take place, mainly depolymerisation and condensation, with a variety of side reactions. Light hydrocarbons like CH_4_, ethane, and propane, and permanent gases like H_2_, CO, and CO_2_ were found in the effluent of reaction. The mentioned products give evidence of the presence of reactions involving radicals formation (cracking reactions). Such reactions are activated as the temperature reaches 220–240 °C. Moreover, oxygen is a promoting agent for radical reactions and its complete scavenging is a consequence of the occurrence of such reactions [19].

The behaviour greatly depends on the intrinsic resistance of the alloy to this form of corrosion. The pitting potential increases with the pitting resistance equivalent index (PRE) of alloys, depending on composition of the stainless steel (mainly chromium and molybdenum). The resistance of AISI 304L steel—having PRE 18—is not enough to prevent the initiation of the attack. Data indicate a certain risk of corrosion in presence of oxygen for a prolonged period. Thus, it appears more conservative to select stainless steel grades with suitable molybdenum content.

Furthermore, the pitting potential also depends on the properties of the protective film. The weight loss measured during tests can be related to colour variations of the specimens after exposure, which is strictly related to the thickness and protectiveness of the passive film. Eish et al. [20] reported that the semi-transparent oxide films become coloured after thermal oxidation at temperatures higher than 200 °C. At temperatures lower than 400 °C, the films are generally too thin for permitting an evaluation of differences in the elements concentration using XPS technique. Trasatti et al. [21] reported that thermal oxidation in the range 150 to 300 °C in air substantially modifies the nature of the passive film of AISI 304L, thus creating conditions that favour nucleation and development of localized corrosion. However, during batch liquefaction, the film develops by chemical oxidation under complex and humid exposure, either on the part placed in the gas phase or immersed. The tests on U-bend specimens didn’t show the clear presence of SCC, but only some micro-defects originated in hardened zones.

After SSR tests, all the specimens showed the presence of cracking that reduced the ductility parameters (see Table 1), but no significant changes of the stress/strain curves were noticed in both the elastic and plastic fields. The cracking was limited to the necking cone in the zone where the material undergoes considerable plastic deformations. Therefore, the phenomena of SCC observed is rather mild, despite the chemical analysis of the aqueous phase indicating a remarkable concentration of chlorides.

The SCC of austenitic stainless steels has been well known since 1930s [22]. Extensive testing has been performed in diluted neutral chloride solutions showing the effect of the critical environmental parameter. Since 1980, Gordon [23] and Speidel [24] showed a quite extensive compilation of SCC data for austenitic stainless steels, including Type 304L and 316L, in term of critical domain of chloride and oxygen concentrations. The importance of oxygen concentration and, hence, potential on the SCC susceptibility was clearly stated. Thus, it can be assumed that at temperatures in the range of 250 to 350 °C and oxygen concentration of 1 ppm, chloride concentrations of 10 ppm can even induce SCC on highly stressed elements.

The absence of clear evidence of SCC can be attributed to some features of batch process. As already observed for the risk of localized corrosion, the duration of the batch conversion process could be too short to promote SCC onset. Furthermore, as previously observed, the oxygen depletion occurs at an early time, modifying corrosion potential. Such a variation can bring the potential to much less critical ranges than in aerated water, reducing the risk of SCC.

**Figure 8:**
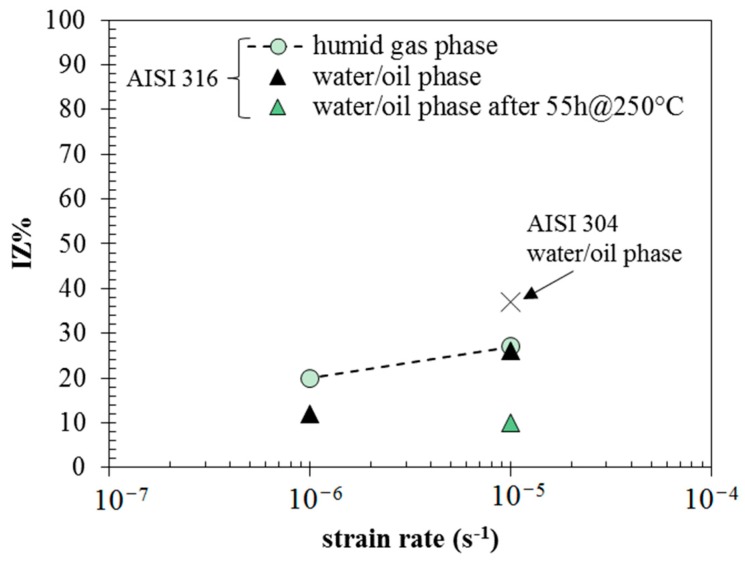
Embrittlement index from reduction of area as a function of strain rate.

Plastic straining at very low strain rates generally enhances environmentally assisted cracking phenomena [25,26,27,28]. The SCC indexes mark the effects of the strain rate, the exposure time, and the position of the specimen in the autoclave (Figure 8). The embrittlement index decreases as the specimen is placed in the bottom of the autoclave, and the strain rate decreases from 10^−5^ s^−1^ to 10^−6^ s^−1^. This seems to be in contradiction with the principles of chloride SCC of austenitic stainless steels, which state higher susceptibilities at 10^−6^ s^−1^ strain rate compared to 10^−5^ s^−1^. The effect of the strain rate is hindered by the primary effect of the extent of liquefaction reaction. In the test at 10^−5^ s^−1^ strain rate performed 55 h after reaching the temperature of 250 °C, the embrittlement index falls to 10. This effect can be attributed to the decrease in the oxygen content, but it should also be noted that an organic deposit forms during prolonged exposure. Therefore, the risk of SCC tends to decrease over time during the bio-oil production process. Actually, the role of organic substances that constitute the bio-oil has to be further demonstrated, but the results of SSR tests carried out at 10^−6^ s^−1^ evidenced higher indexes in the upper part (20) rather than in the lower part (12), meaning that a certain shielding effect can also be hypothesized (Figure 8). The presence of such organic deposits was also evidenced on the surface of U-bend specimens. The EDS analysis before pickling identified a large number of elements, mainly Cl, Si, Al, Na, K, and Ca. Sulphur was not clearly identified because of overlapping with the peak of the molybdenum in base metal. Sporadic presence was determined for deposits of sodium chloride, potassium, and calcium phosphate. Relevant concentrations of amides in bio-oil was evidenced by means of gascromatographic analysis with mass detection (GC-MS), as reported elsewhere by Chiaberge et al. [15]. Amides were also measured in ethyl acetate solvent used for washing the specimens. The adsorption of such organic substances on the metallic surface could play a crucial role in terms of corrosion mitigation, having inhibiting properties as from previous woks by Di Iorio et al. [29], Fang [30], Ghareba and Omanovic [31], and Yu [32].

## 4. Materials and Methods

### 4.1. Materials

The corrosion tests were performed on AISI 316L and 304L stainless steels, purchased in the form of a 1000 × 2000 mm^2^ sheet with 2.5 mm thickness or 6 mm diameter bar. Table 2 shows the alloy compositions.

The steel microstructure was verified by oxalic acid etching, according to Practice A of ASTM A262 standard for detecting susceptibility to intergranular attack. Both steels show a “step” microstructure without carbide precipitation at grain boundary.

### 4.2. Environment

The corrosion tests were carried out during the liquefaction of a Simulated Organic fraction of MSW (SMSW). The same SMSW composition was used for all the tests. The charge was prepared by cutting and mixing different foods in order to reproduce the same average content of protein, salts, carbohydrates, lipids, water, etc., of a typical organic fraction of MSW. It was packaged and frozen at −20 °C in different amounts and thawed just before use. Table 3 shows the elementary composition of the dry matter of the charge, the dry matter is 30% of the total weight, being the complement to 100% water.

The liquefaction reaction was performed in batch reactors at the temperatures of 240–250 °C or 300 °C, from 55 up to 264 h. Pressure ranges between 40 and 100 bar. As mentioned in the introduction, the bio-oil TAN is influenced by the operating conditions of liquefaction and characteristics of the charge. The TAN number is statistically proportional to the lipid fraction of the charge and with a same charge, composition tends to slightly decrease with higher temperatures and contact time. In the temperature range investigated from 240 to 310 °C, with a charge containing 29% of lipid content, the conversion of the SMSW produces bio-oils with TAN of about 85 mg KOH/g.

### 4.3. Corrosion Tests and Stress Corrosion Cracking Tests

**Figure 9:**
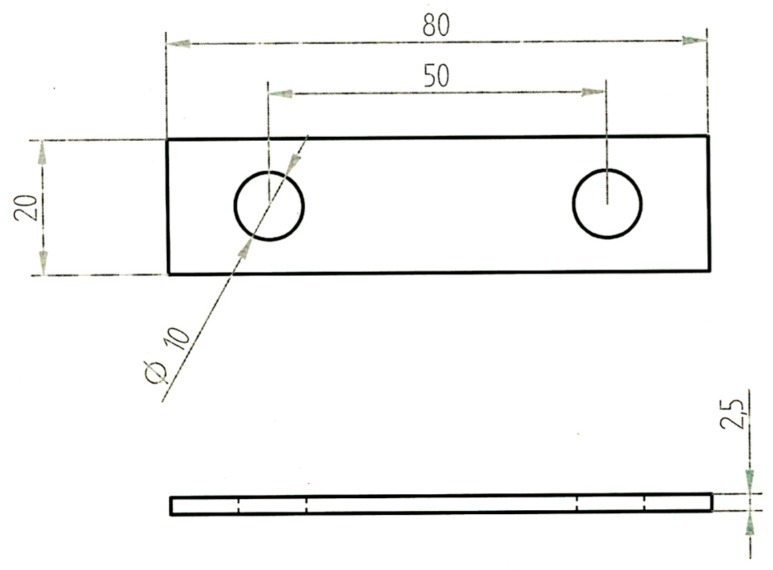
U-bend specimens for corrosion and constant-load stress corrosion cracking tests (all measurements are in millimeters).

U-bend specimens were prepared according to type A of ASTM G30-97 (Figure 9). They were polished with emery papers up to 1200 grit, degreased in acetone, dried, and weighed with an analytical scale with accuracy of 0.1 mg. Afterward, they were pre-formed to a U-shape by 10 mm diameter mandrel and loaded by bolt.

**Figure 10:**
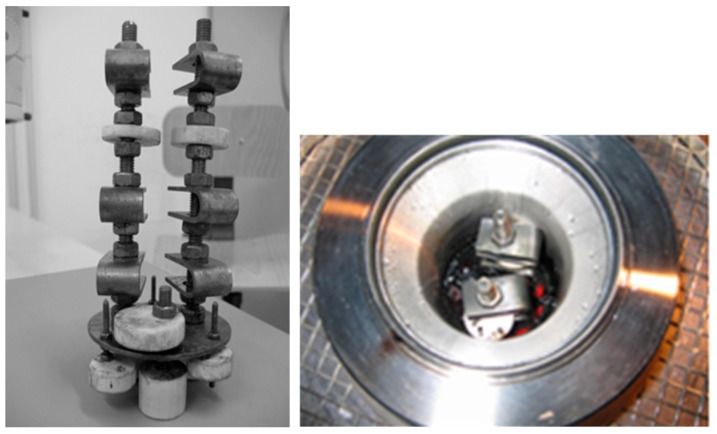
U-bend specimens after the test at 250 °C after 55 hours exposure.

The sample holder consists of a lower plate with three threaded columns with a diameter of 8 mm, made of the same type of stainless steel of specimens in order to avoid any galvanic coupling. The specimens were tightened on two of these columns on different levels in order to expose the material to different zones of the reactor. PTFE sheaths were mounted in order to achieve electrical insulation between the specimens and the autoclave. Other PTFE spacers were mounted on the plate and on the upper end for regulating the system centring (Figure 10). Before the tests, the electrical insulation between holder and test vessel was checked.

The reactor (autoclave in Hastelloy C276) had a volume of 680 mL and was filled with about 300 g of SMSW, in order to occupy 2/3 of the total volume. During tests, the specimens at the top of the holder were in contact with the gas phase, while the others were immersed in the oil/water phase.

After testing, the autoclave was cooled to 40 °C and then it was opened in order to extract the specimens. The specimens were firstly cleaned with ethyl acetate, then ultrasonically cleaned in acetone before the observation of surfaces by means of optical and scanning electron microscope (SEM). Later, the specimens were further cleaned in hot sodium hydroxide and nitric acid (50%) at a temperature of 60 °C in order to dissolve carbonaceous residues. Afterward, the specimens were dried and weighed to evaluate the weight loss. In order to estimate the weight loss due to the cleaning procedure, some untested specimens were pickled by using the same cleaning procedure. The weight loss of these specimens was negligible.

**Figure 11:**
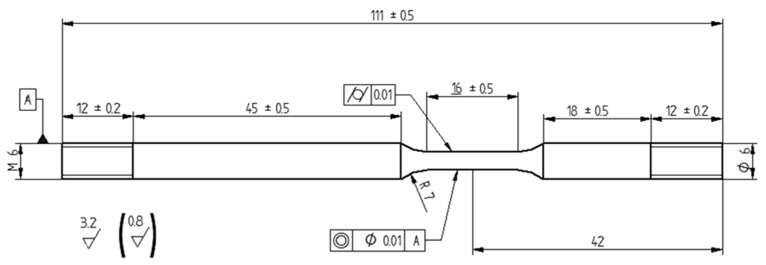
Geometry of SSR specimen (all measurements are in millimeters).

The SSR tests were performed according to ASTM G129-00 and EN ISO 7539-7, on cylindrical specimens machined from stainless steel bars (Table 2, Chemical composition (wt %) of steels) with an asymmetric geometry (Figure 11), in order to expose the gauge length in the upper or lower position of the autoclave.

The specimens were loaded under constant elongation rate until break, at initial strain rates of 10^−5^ and 10^−6^ s^−1^ referred to gauge length. The insurgence of SCC was evaluated by comparing the results of test performed into the aggressive environment to the result in air.

The tests were performed in an autoclave made of Hastelloy C and featuring an inner volume of about 0.5 L during the liquid phase test. The specimen was positioned with the gauge length in the lower region of the reactor, immersed in about 430 g of SMSW. During testing in the gas phase, the gauge length was in the upper zone of the reactor and the SMSW volume was limited to 300 g.

The loading was applied after the charge inside the autoclave reached the target testing temperature during the early stage of SMSW conversion. The test then continued until specimens break occurred, typically for about five hours at 10^−5^ s^−1^ strain rate and 55 h at 10^−6^ s^−1^ strain rate. Tests at 10^−5^ s^−1^ were also performed after the charge conversion process in order to evidence the effect of the modification of the environment on the function of the extent of the conversion reaction.

After the rupture of the specimen, the autoclave was cooled down to about 60 °C before discharging the residual pressure. The gases coming out were treated through an abatement solution of sodium hypochlorite. Then the autoclave was purged with nitrogen and the specimens were taken out. The specimens were cleaned in ethyl acetate and they were measured in order to estimate the total elongation and reduction of area. Finally, the fracture surface was observed by means of optical microscope and scanning electron microscope (SEM).

## 5. Conclusions

The paper investigates the corrosion and stress corrosion cracking behaviour of AISI 316L and AISI 304L stainless steels in environments typical of the sub-critical liquefaction processes for bio-oil production from the organic fraction of MSW. Very low corrosion rates strictly dependent upon time and temperature were noticed due to the formation of passive film of chromium-rich oxide. This protective film is stable in a wide range of pH. Despite the high chloride concentration of the water phase and high temperature, pitting corrosion was not clearly noticeable, and the initiation of pitting was only observed on AISI 304L steels, without any significant propagation. The low pitting susceptibility can be mainly ascribed to the nature of batch process, which promotes the formation of reducing conditions. However, critical conditions during process transients can occur.

SCC was not observed on U-bend specimens under constant loading. Small cracks confined in the necking cone of specimens were noticed after SSR tests, meaning that SCC phenomena take place only under dynamic loading in the plastic field. The micro-cracks depth was less than 150 μm. Despite the high content of chlorides in the aqueous phase (close to 5000 mg/L), the oxygen depletion and the formation of a deposit of organic products reduce the risk of SCC during the liquefaction process. The embrittlement indexes calculated after SSR tests confirmed a slightly better resistance to SCC of AISI 316L steel compared to AISI 304L.

## Figures and Tables

**Table 1:** Values of SCC indexes calculated after SSR tests at 250 °C during liquefaction of SMSW.

Material	Strain Rate (s^−1^)	Position Specimen’s Gauge Length	Waiting Time (h) *	IA%	IZ%
AISI 316L	10^−5^	Liquid phase	0	4	26
			55	21	10
		Gas phase	0	25	27
	10^−6^	Liquid phase	0	21	12
		Gas phase	0	21	20
AISI 304L	10^−5^	Liquid phase	0	38	37

* Specimen immersed into the testing environment before loading application.

**Table 2:** Chemical composition (wt %) of steels.

Steels	Form	C	Mn	Si	P	S	Cr	Ni	Mo	N	Cu	YS * (MPa)	UTS ** (MPa)
AISI 316L	sheet	0.026	0.9	0.32	0.03	0.001	16.75	10.30	2.09	0.048	0.39	268	583
	bar	0.03	1.45	0.34	0.029	0.03	16.6	10.5	2,01	0.05	0.32	609	735
AISI 304L	sheet	0.026	1.77	0.33	0.026	0.001	18	8.07	0.19	0.06	0.18	265	612
	bar	0.044	1.36	0.34	0.032	0.022	18.3	8.1	0.24	0.08	0.33	611	843

* YS = Yield Strength; ** UTS = Ultimate Tensile Strength.

**Table 3:** Average composition of the SMSW.

Charge	N	C	H	S	O	Ash
OS-MSW typical composition (wt %)	2–4	40–60	7–10	0.1–0.2	23–35	2–7
SMSW—present study composition (wt %)	4.1	50.6	7.9	0.2	30.1	5.7
